# Mitochondria‐Associated Endoplasmic Reticulum Membranes in Human Health and Diseases

**DOI:** 10.1002/mco2.70259

**Published:** 2025-06-27

**Authors:** Yong Liu, Zi‐Hui Mao, Junwen Huang, Hui Wang, Xiao Zhang, Xin Zhou, Yue Xu, Shaokang Pan, Dongwei Liu, Zhangsuo Liu, Qi Feng

**Affiliations:** ^1^ Department of Nephrology The First Affiliated Hospital of Zhengzhou University Zhengzhou University Zhengzhou China; ^2^ Research Institute of Nephrology Zhengzhou University Zhengzhou China; ^3^ Department of Integrated Traditional and Western Nephrology The First Affiliated Hospital of Zhengzhou University Zhengzhou China; ^4^ Henan Province Research Center for Kidney Disease The First Affiliated Hospital of Zhengzhou University Zhengzhou China; ^5^ Tianjian Laboratory of Advanced Biomedical Sciences Academy of Medical Sciences Zhengzhou University Zhengzhou China; ^6^ Hebi Branch of Henan Academy of Sciences Hebi China

**Keywords:** mitochondria, endoplasmic reticulum, mitochondria‐associated endoplasmic reticulum membranes, human diseases

## Abstract

As fundamental units of life activities, cells exhibit a high degree of structural refinement and functional specialization, forming the cornerstone of life complexity. Compartmentalization within cells is pivotal for maintaining the orderly progression of intracellular biochemical processes. Cellular compartments constitute the enclosed regions within the cytoplasm of all eukaryotic cells and are typically surrounded by a single or double layer of phospholipids, and include major organelles, such as the endoplasmic reticulum (ER) and mitochondria. Compartmentalization enables organelles to maintain distinct environments in terms of space, physics, and chemistry, thereby increasing their functionality. Human health is closely associated with cellular organelle homeostasis, and organelle dysfunction affects disease pathogenesis. In contrast to isolated cellular compartments, organelles are interdependent and communicate via membrane contact sites, with close membrane contact between the ER and mitochondria, forming mitochondria‐associated ER membranes (MAMs), which are involved in multiple cellular functions and whose integrity and function are essential for cellular homeostasis, with dysfunction implicated in various diseases. Investigating MAMs structure, function, and disease‐state alterations informs mechanisms and developing therapies. This article reviews the discovery, structure, function, and research progress of MAMs in human systemic diseases and cancer and explores their potential as therapeutic targets.

## Graphical abstract

MAM dysfunction is considered as an initiator or contributor in the progression of various diseases such as nervous system diseases, circulatory system diseases, digestive system diseases, locomotor system diseases, reproductive system diseases, endocrine system diseases, urinary system diseases, respiratory system diseases and cancer.

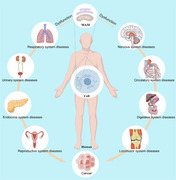

## 1 Introduction

Eukaryotic cells are compartmentalized into membrane‐bound organelles that isolate molecules and proteins to increase the speed of specific processes and ensure that various cellular reactions occur in defined spaces [1, 2]. Mitochondria and endoplasmic reticulum (ER) are two key organelles that play central roles in the regulation of cellular physiology and pathology. ER, the largest membrane‐bound organelle, is involved in multiple cellular functions, including protein synthesis and modification, lipid metabolism, calcium ion (Ca^2+^) homeostasis, secretion pathways, and membrane structure formation [3, 4]. Owing to their highly dynamic nature, mitochondria participate in the regulation of energy metabolism, reactive oxygen species (ROS) production, inflammatory responses, and cell death [5, 6]. The highly specialized division of labor, precise coordination, and close physical contact between organelles form a dynamic network that not only facilitates the rapid exchange of materials and information but also facilitates flexible regulation of life activities in response to environmental changes [7]. However, metabolic compartmentalization introduces spatial discontinuities that pose challenges. To address this, cells use various strategies to coordinate the metabolic flux between different organelles, such as membrane‐located transport proteins, nonvesicular transport pathways, and localized modulation of signal transduction [8]. Communication between the ER and mitochondria is crucial for maintaining cellular homeostasis and coordinating diverse biological activities. A deeper understanding of mitochondrial ER communication holds promise for revealing the mechanisms of cellular function, development and progression of diseases, and the development of new therapeutic strategies.

**FIGURE 1:**
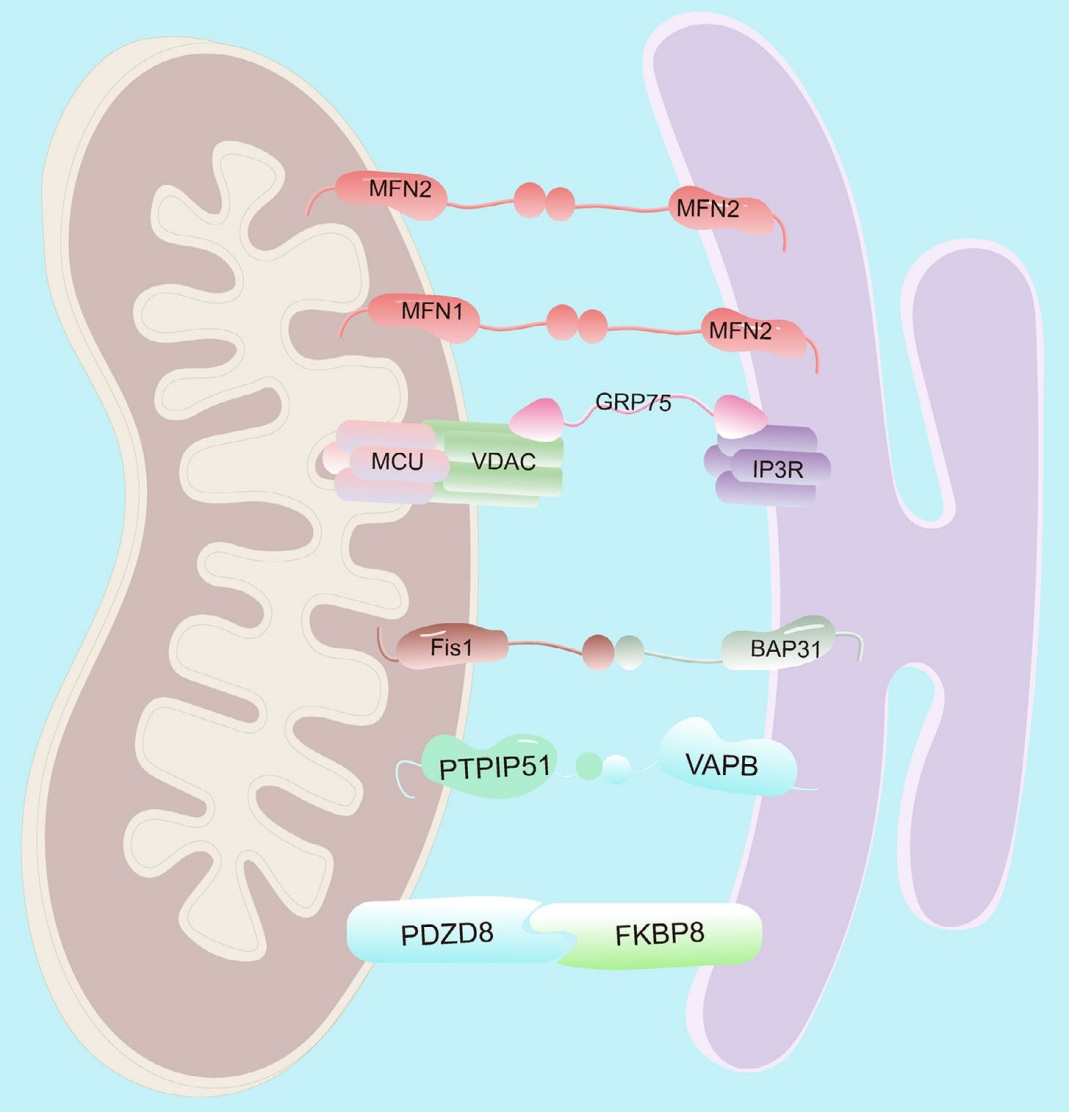
Structural architecture of MAMs. MAMs are intricate structures comprising the mitochondrial outer membrane, the ER membrane, and a diverse array of associated proteins. The formation and maintenance of MAMs are facilitated by several molecular connectors, including the following: (1) MFN dimers: Homodimers and heterodimers of mitofusin 1 (MFN1) and mitofusin 2 (MFN2), which are GTPases located on the outer mitochondrial membrane, play pivotal roles in tethering the two organelles; (2) Inositol IP3R–GRP75–VDAC complex: This macromolecular complex bridges the ER and mitochondria, facilitating calcium signaling and metabolic coupling; (3) Fis1/BAP31 interaction: The interaction between Fis1, a tail‐anchored protein on the mitochondrial outer membrane, and BAP31, an ER‐resident protein, contributes to the structural integrity and functional regulation of MAMs. (4) VAPB/PTPIP51 interaction: The interaction between VAPB on the ER and PTPIP51 on the mitochondrial outer membrane is crucial for tethering and regulating lipid exchange between the two organelles. (5) PDZD8/FKBP8: The interaction between FKBP8 on the ER and PDZD8 on the mitochondrial outer membrane mediates the close apposition of the ER and mitochondria and facilitates lipid transfer between them. IP3R: Inositol 1,4,5‐trisphosphate receptor; GRP75: Glucose‐regulated protein 75; VDAC: Voltage‐dependent anion channel; MCU: Mitochondrial calcium uniporter; BAP31: B cell receptor associated protein 31; Fis1: Mitochondrial fission 1 protein; PTPIP51: Protein tyrosine phosphatase‐interacting protein‐51; VAPB: Vesicle‐associated membrane protein‐associated protein‐B; PDZD8: PDZ domain containing 8; FKBP8: FK506 binding protein 8.

**FIGURE 2:**
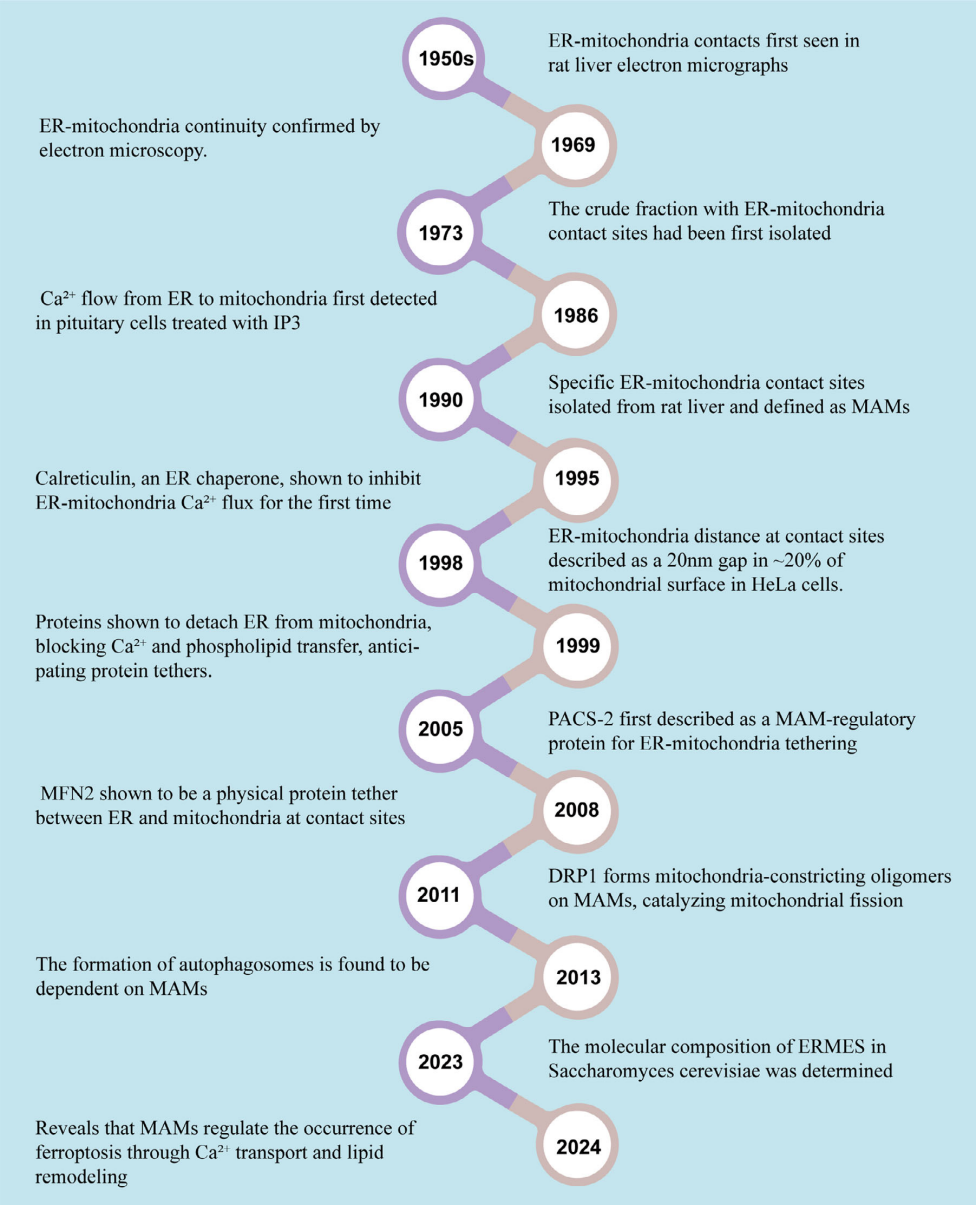
Timeline of key developments in the field of MAMs. Since the 1950s, the contacts between the ER and mitochondria have been extensively studied. In the 1950s, ER‒mitochondrion contacts were first observed in electron micrographs of the rat liver. In 1969, electron microscopy confirmed the continuity between the ER and mitochondria. In 1973, Lewis et al. isolated the first crude fraction containing ER‒mitochondria contact sites. A significant breakthrough occurred in 1986 when Ca^2^⁺ flow from the ER to the mitochondria was first detected in pituitary cells treated with IP3. In 1990, specific ER‒mitochondrion contact sites were isolated from rat livers and defined as MAMs. In 1995, calreticulin, an ER chaperone, was shown for the first time to inhibit Ca^2^⁺ flux between the ER and mitochondria. By 1998, the distance between the ER and mitochondria at contact sites was described as a 20 nm gap, which is present on approximately 20% of the mitochondrial surface in HeLa cells. In 1999, proteins that could detach the ER from mitochondria, blocking Ca^2^⁺ and phospholipid transfer, which led to the discovery of protein tethers, were identified. In the 21st century, research in this field advanced significantly. In 2005, PACS‐2 was the first protein described as a MAM‐regulatory protein because of its role in mediating ER–mitochondria tethering. In 2008, mitofusin‐2 was identified as a physical tether between the ER and mitochondria at contact sites. By 2013, it was discovered that the formation of autophagosomes was dependent on MAMs. In 2023, the molecular composition of the ER–mitochondria encounter structure (ERMES) in *Saccharomyces cerevisiae* was determined, and a molecular model of ERMES was established. Most recently, in 2024, MAMs were shown to regulate the occurrence of ferroptosis through calcium ion transport and lipid remodeling.

Mitochondria‐associated ER membranes (MAMs) are transient structural domains in which the outer mitochondrial membrane (OMM) is in close proximity or in direct contact with the ER membrane (Figure 1) [9, 10, 11]. The discovery of MAMs represents an important milestone in the field of cell biology, indicating the complex and intricate interactions between two key organelles, the ER and mitochondria (Figure 2). Although the possibility of a tight association between mitochondria and the ER was first suggested in 1956 via electron microscopy in rat liver cells [12], it took many years before this interaction was widely accepted. By the early 1980s, most cell biologists still considered the idea of physical contact between the ER and mitochondria an artifact [13]. In 1990, Vance used gradient centrifugation and subfractionation techniques to isolate MAMs and defined the specific contact site between the ER membrane and mitochondria as MAMs [14]. Although initially mentioned in limited citations, this paper is now recognized as pioneering work in describing MAMs as essential for phospholipid biosynthetic enzyme activity. Another study has shown that when the ER approaches the mitochondria within nanometers, mitochondria–ER contacts form on approximately 20% of the mitochondrial surface [15]. The extent and length of these contacts are influenced by cellular stress conditions [16].

Electron tomography indicates that the ER and mitochondria are connected by slender tether‐like structures with widths of 9–30 nm depending on the type of ER (smooth or rough) [17]. Electron microscopy observations have shown that MAMs are heterogeneous in structure and can be classified into three types based on the size of the contact area between the ER and mitochondria: Type I, with approximately 10% contact area, where mitochondria can move independently; Type II, with an increased contact area of 50%, which is commonly found in brown adipose tissue; and Type III, where the ER completely envelops the mitochondria [18]. The presence of MAMs not only facilitates communication between the ER and mitochondria but also endows them with new properties and functions.

To date, the existence of MAMs has been fully confirmed using various techniques, including live imaging, new electron microscopy methods, and subcellular fractionation [19, 20]. MAMs are protein‐ and lipid‐rich structures, and proteomic analysis has identified hundreds to thousands of proteins in MAMs from various species and tissues [21]. As a bridge between the ER and mitochondria, MAMs rely primarily on proteins to perform their functions and play a key role in regulating cellular physiology and metabolic homeostasis [22]. However, MAMs are highly tissue specific, and the proteins enriched in MAMs from different organs differ substantially [23]. These MAM proteins can be categorized into three groups: those that are exclusively located in MAMs; those that are present in other organelles as well as in MAMs; and those that transiently relocate to MAMs upon various cellular stimuli [24]. MAMs are crucial for maintaining cellular homeostasis and determining the fate of cells under stress conditions [25]. Their protein interactions are involved in various processes such as Ca^2+^ homeostasis, lipid synthesis and transport, regulation of mitochondrial morphology, ER stress (ERS), apoptosis, and autophagy.

Recent research has made considerable progress in understanding the role of MAMs in cellular stress responses, development and progression of diseases, and their potential as therapeutic targets. The discovery and study of MAMs have improved our understanding of the mechanisms of intracellular material exchange and signal transduction and have provided important scientific evidence for the development of new therapeutic strategies. Some studies have suggested that MAMs dysfunction is associated with various metabolic, neurodegenerative, and cardiovascular diseases [26, 27, 28, 29]. This review revisits the development of MAMs research and explores the role of mitochondria–ER communication in health and disease to elucidate cellular adaptive processes and potential therapeutic targets for treating disease.

## 2 Structural and Functional Architecture of MAMs

MAMs represent specialized membrane contact sites that physically bridge the ER and outer OMM [10]. These domains form dynamic protein–lipid interfaces through coordinated molecular interactions, enabling direct interorganellar communication and material exchange [20, 30, 31]. The structural architecture of ER–mitochondrial junctions exhibits remarkable complexity, with multiple protein complexes mediating distinct aspects of interorganellar communication (Table 1). The IP3R1–GRP75–VDAC1 tripartite complex establishes a Ca^2^⁺ transmission channel that synchronizes mitochondrial ATP production with cytosolic Ca^2^⁺ oscillations [90, 91]. Mitofusin 2 (MFN2), dually localized to OMM and ER membranes, regulates membrane proximity through GTPase‐dependent homotypic interactions while coordinating mitochondrial fusion [92, 93]. The vesicle‐associated membrane protein B (VAPB)–phosphatidylinositol transfer protein 51 (PTPIP51) complex enhances membrane tethering while directly modulating lipid transfer [33], autophagosome formation [94], and Ca^2^⁺ homeostasis [95]. Similarly, the Fis1–BAP31 complex facilitates caspase‐8‐mediated apoptotic signaling [35], whereas PDZD8 emerges as a critical regulator of Ca^2^⁺ dynamics [96, 97] and mitochondrial morphology through FKBP8‐dependent cristae remodeling [98], collectively demonstrating functional diversification among MAM tethers.

**TABLE 1:** Major related proteins and functions of MAMs.

Role	Protein	Functions and mechanisms	References
MAMs tethers	MFN2	Physically interacting with MFN1 or MFN2 on OMM to connect the ER and mitochondria	[32]
	VAPB–PTPIP51	Enhances membrane tethering, regulates lipid transfer, autophagosome formation, Ca^2^⁺ homeostasis	[33]
	IP3R1–GRP75–VDAC1	Form a tethering machinery that coordinates Ca^2+^ transfer	[34]
	Fis1–BAP31	Stabilizes ER–mitochondria contact sites, balancing ER–mitochondria communication for Ca^2^⁺ homeostasis, energy metabolism, and apoptotic signaling	[35]
	PDZD8	Represents a sophisticated molecular bridge that coordinates ER–mitochondria communication for Ca^2+^ signaling, lipid metabolism, and organelle dynamics	[36]
Ca^2+^ homeostasis	RyRs	Located on the ER, release Ca^2^⁺ from the ER upon stimulation	[37]
	IP3Rs	Release Ca^2^⁺ from the ER into the cytoplasm upon binding of IP3, and interact with VDAC1 at MAMs to form a Ca^2^⁺ transport channel	[37]
	VDAC1	Forms an efficient Ca^2^⁺ transport channel with IP3Rs at MAMs along the electrochemical gradient	[38]
	GRP75	A chaperone that enhances the interaction between IP3R and VDAC1	[39]
	SERCA	Pumps Ca^2^⁺ from the cytoplasm into the ER to maintain ER Ca^2^⁺ storage	[37]
	IRE1α	Stabilizes IP3R	[40]
	FUNDC1	Stabilizes IP3R	[41]
	VAPB	Influence the interaction between IP3R and VDAC1	[42]
	PTPIP51	Influence the interaction between IP3R and VDAC1	[42]
Lipid synthesis and transport	PSS	Synthesizes PS within the ER	[43]
	PSD	Converts PS to PE in the inner mitochondrial membrane	[43]
	ORP5/8	Mediate nonvesicular transport of PS lipids from the ER to mitochondria	[44]
	MFN2	Binds PS and enhance PS delivery to mitochondria, thereby supporting mitochondrial PE biosynthesis	[45]
	CDS2	Mediates the nonvesicular transport of PS from the ER to the mitochondria	[46]
	VAPB	Interacts with PTPIP51to mediate phospholipid transfer between the ER and mitochondria	[33]
	PTPIP51	Interacts with VAPB to mediate phospholipid transfer between the ER and mitochondria	[33]
	Lipocalin 2	A PA binding protein recruited to the MAMs during inflammation and metabolic stimulation	[47]
	ERK	Recruits ER–plasma membrane tethers and lipid transfer proteins such as E‐Syt1 at ER–mitochondria contact sites for mitochondrial lipid transport and respiration	[48]
	E‐Syt1	Recruited by PERK at ER–mitochondria contact sites for mitochondrial lipid transport and respiration.	[48]
	Caveolin‐1	Promote the transfer of cholesterol at mitochondrial–ER contact sites	[49]
	GRAMD1C	Mediates cholesterol transport between the ER and mitochondria, while inhibiting mitochondrial bioenergetics	[50]
Mitochondrial homeostasis	DRP1	Promotes mitochondrial fission by forming ring‐like structures at constriction sites, recruited by MAMs proteins	[51]
	Sig‐1R	Interacts with MFN2 at MAMs, promoting MFN2 oligomerization and stabilizing ER–mitochondria contact	[52]
	MFN1/2	Mediate mitochondrial outer membrane fusion	[53]
	STX17	Recruits DRP1 to MAMs for fission regulation	[54]
	DsbA‐L	Facilitates DRP1 recruitment to MAMs for fission	[55]
	PACS‐2	Mediates DRP11 translocation to MAMs for fission	[56]
	Fis1	Promotes fission in the absence of DRP1 by inhibiting mitochondrial fusion	[57]
	REEP5	Interacts with MFN1/2 to regulate mitochondrial “hitchhiking” on tubular ER, ensuring even mitochondrial distribution	[58]
	FUNDC1	Interacts with ULK1 via Lon protease accumulation at MAMs, inducing mitophagy through chaperone activity	[59]
ER stress	Sig‐1R	Enhances cell survival during early ER stress by stabilizing IRE1α dimerization and activation at MAMs as an adaptive regulator of the UPR	[60]
	IRE1α	Splices XBP1 mRNA, upregulates folding and degradation enzymes	[61]
	MITOL	Enriches in MAMs during ERS, thereby promotes IRE1α modification and inhibits its excessive activation	[62]
	PERK	Phosphorylates eIF2α to suppress global protein synthesis, limit ER protein influx, and selectively upregulate stress‐response proteins to mitigate ERS	[63, 64]
	MFN2	Act as upstream inhibitors of PERK activity and maintain ER homeostasis by directly interacting with and inhibiting PERK activation	[65]
Apoptosis	IP3R	Excessive activation of IP3R under apoptotic stimuli leads to the transfer of excessive Ca^2^⁺ from the ER to the mitochondria	[66]
	Akt	Phosphorylates IP₃R3 at MAMs, suppressing Ca^2^⁺ transfer and blocking apoptosis	[67]
	Bcl‐xL	Inhibits IP₃R‐mediated Ca^2^⁺ release, dampening Ca^2^⁺‐dependent apoptosis	[68, 69]
	Bax	Translocates to mitochondria via MAMs, inducing cytochrome *c* release and mPTP opening through mitochondrial Ca^2^⁺ overload	[70]
	Fis1	Binds to Bap31, promotes ER–mitochondrial tethering, recruits and activates procaspase‐8, and initiates early apoptotic events	[71]
	Bap31	Stimulates ER Ca^2^⁺ release, exacerbating mitochondrial Ca^2^⁺ overload and apoptosis	[72]
	DRP1	Colocalize with Bax at mitochondria during apoptosis, mediating membrane permeabilization and fragmentation	[73]
Autophagy	ULK1	Translocates to MAMs upon starvation, forming PI3K complex with Atg14/BECN1 to generate PI3P for phagophore initiation	[74, 75]
	Atg5	Extend the autophagosome membrane and promote the transmembrane transport of LC3 to drive the maturation of autophagosomes.	[76]
	Atg2A	Transferred to MAMs via TOM40/TOM70, interacts with Atg9, promotes phagophore expansion and autophagosome enlargement, enhancing autophagic flux	[77]
	STX17	Interacts with Atg14 at MAMs to promote autophagosome–lysosome fusion, while mitochondrial–ER contact disruption impairs Atg14 puncta formation	[78]
	AMPK	Activated by BECN1, promoting local autophagy	[79]
	PML	localizes to MAMs in a Ca^2+^‐dependent manner in P53‐driven cells and inhibits autophagy	[80]
	MFN2	Phosphorylated by AMPK at MAMs, promoting autophagy under energy stress	[81]
	RTN3L	A Rab32 effector, drives selective protein autophagy at MAMs	[82]
Ferroptosis	MFN2	Deletion impairs MAMs function, potentially enhancing ferroptosis	[83]
	GRP75	Phosphorylation by PKA (via AKAP1) sequesters GRP75 outside mitochondria, activating Nrf2‐mediated antioxidant defense and inhibiting ferroptosis	[84]
	VDAC1	Interact with IP3R to mediate the influx of Ca^2^⁺ and regulate lipid peroxidation	[85]
	AKAP1	Anchoring PKA to MAMs inhibits ferroptosis.	[84]
	PERK	Regulate Ca^2^⁺ and lipid metabolism, affecting MAMs function.	[86]
	ACSL4	Promote lipid peroxidation and exacerbate ferroptosis.	[86]
Inflammation	NLRP3	Depend on MAMs to form NLRP3 inflammasomes	[87]
	STING	Activation of Ca^2^⁺ signaling mediated by MAMs, while feedback inhibition of MAMs function to maintain mitochondrial homeostasis	[88]
	VDAC2	Negatively regulates inflammation by inhibiting MAMs formation via interaction with STING	[89]

Beyond their structural constituents, MAMs integrate an extensive repertoire of functional regulators that orchestrate their biological activities. These regulatory components can be categorized into several groups: calcium signaling machinery, including transient receptor potential vanilloid 1 (TRPV1, facilitating ER‐to‐mitochondria Ca^2^⁺ flux) [99], Parkinson disease protein 7 (PARK7/DJ‐1, maintaining IP3 receptor homeostasis) [100], and Sigma‐1 receptor (Sig‐1R, stabilizing IP3R1 under stress conditions) [101, 102]; lipid metabolic enzymes such as dihydroceramide desaturase 1 (generating ceramides) [103] and mitoguardin‐2 (mediating phosphatidylserine [PS] transport) [104, 105]; mitochondrial quality control systems involving dynamin‐related protein 1 (DRP1, regulating ER contact site‐associated fission) [106] and autophagy and beclin 1 (BECN1) regulator 1 (AMBRA1, recruiting autophagy machinery) [107, 108]; and cell death mediators such as Bcl‐2‐associated X protein (Bax, governing OMM permeabilization) [109]. These components collectively enable MAMs to spatiotemporally integrate organelle dynamics, metabolic adaptation, and cell fate determination.

The functional sophistication of MAMs arises from their unique lipidomic landscape, which cooperates with protein networks to regulate membrane organization. MAMs exhibit 40–60% higher cholesterol content than bulk ER membranes, enriched with phosphatidylethanolamine (PE), phospholipids phosphatidylserine (PS), and sphingolipids that form lipid raft microdomains [110, 111]. These domains serve as critical platforms for phospholipid biosynthesis, particularly PE and PS synthesis, where intermediate metabolites like phosphatidic acid (PA) accumulate to fuel dynamic lipid remodeling [47, 112, 113, 114]. The high cholesterol and sphingolipid content facilitates the formation of lipid raft microdomains, creating a unique biophysical environment that promotes protein clustering and signal transduction cascades [115, 116]. Importantly, this lipid‐protein interplay establishes a spatiotemporally organized platform that couples metabolic flux with interorganelle communication, positioning MAMs as central hubs for lipid‐mediated signaling and membrane remodeling. The spatial segregation of these lipid species not only dictates local membrane properties but also ensures precise regulation of signaling microdomains, positioning MAMs as central hubs for lipid‐mediated cellular regulation.

It is important to emphasize that MAMs, as specialized functional contact sites between the ER and mitochondria, exhibit remarkable spatiotemporal plasticity rather than fixed structures, dynamically adapting to cellular demands through structural and functional reorganization [117, 118]. The morphology, contact area, and proteomic composition of MAMs undergo continuous adaptation in response to cellular states—for instance, during mitochondrial fission, ER tubules envelop mitochondrial constriction sites and recruit DRP1 through ER‐resident proteins including C1q/tumor necrosis factor‐related protein 1 (CTRP1), reticulon‐4 (RTN4)4, and cytoskeleton‐linking membrane protein 63 (CLIMP‐63), to mediate mitochondrial fission [119, 120, 121]. The ER–mitochondria distance demonstrates dynamic regulation, as evidenced by Sun et al.’s [122] work showing its correlation with mitochondrial size and modulation by metabolic intermediates including glucose, glutamine, phosphoenolpyruvate, citrate, and free cholesterol. This interorganelle distance is further regulated by specific protein interactions, notably during myocardial ischemia/reperfusion injury where diaphanous‐related formin 1 (DIAPH1) modulates SR/ER‐mitochondrial proximity through MFN2 interactions [123]. The lipid composition of MAMs exhibits metabolic responsiveness, with membrane fluidity and signaling efficiency being modulated under pathological conditions—for example, autophagic stimuli induce cardiolipin (CL) translocation from mitochondrial inner membranes to MAMs where it interacts with AMBRA1 to promote autophagosome formation [124]. Collectively, MAMs maintain structural, compositional, and functional homeostasis through dynamic recruitment of protein complexes, lipid remodeling, and cross‐talk between signaling pathways. This plasticity enables MAMs to serve as regulatory hubs that coordinate cellular metabolism, stress responses, and pathological adaptations. However, dysregulation of these dynamic processes may contribute to metabolic disorders, neurodegeneration, and oncogenesis through disrupted cellular homeostasis.

## 3 The Physiological Function of MAMs

**FIGURE 3:**
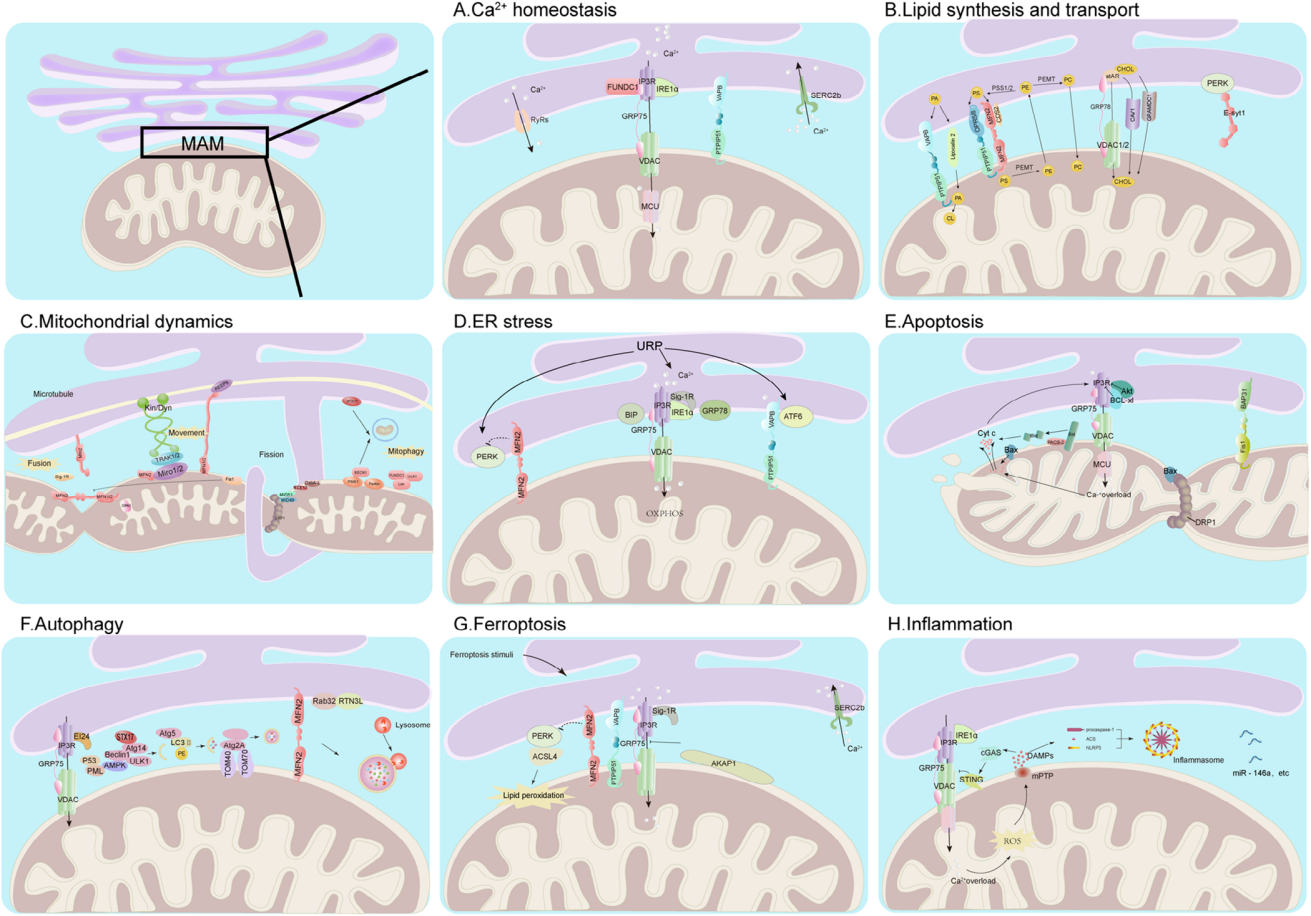
The physiological functions of MAMs. MAMs mediate Ca^2+^ homeostasis (A), Lipid metabolism synthesis and transport (B), Mitochondrial dynamics (C), ER stress (D), Apoptosis (E), Autophagy (F), Ferroptosis (G), and Imitochondrial fission factor (Mff)nflammation (H).

MAMs play crucial roles in various physiological processes, including regulation of Ca^2+^ homeostasis, lipid synthesis and transport, mitochondrial dynamics, autophagy, inflammation, and cell death (Figure 3).

### 3.1 Ca^2+^ Homeostasis

Ca^2+^ is an important intracellular signaling molecule involved in multiple physiological processes, such as muscle contraction, cell secretion, and gene expression regulation. The ER and mitochondria are the main participants in cellular Ca^2+^ homeostasis. MAMs serve as platforms for Ca^2+^ transport between the ER and mitochondria, coordinating ER–mitochondrial interactions to avoid adverse effects caused by Ca^2+^ overload or depletion [125]. The ER is the primary Ca^2+^ storage site in the cell; upon stimulation, Ca^2+^ is released from the ER through ryanodine receptors (RyRs) or inositol‐1,4,5‐trisphosphate receptors (IP3Rs), or taken up by the sarcoplasmic/ER Ca^2+^‐ATPase (SERCA) to regulate cellular Ca^2+^ homeostasis [37]. A portion of the released Ca^2+^ is taken up by the mitochondria through MAMs. After uptake, mitochondria regulate their metabolic activities and buffer changes in cytosolic Ca^2+^ concentrations, preventing Ca^2+^ overload and potential damage to the cell [126, 127, 128].

The mitochondrial calcium uniporter (MCU), which has low affinity for Ca^2+^, requires high Ca^2+^ concentrations for Ca^2+^ influx into mitochondria [129]. In MAMs, the interaction between IP3Rs on the ER and the voltage‐dependent anion channels (VDAC1) on the mitochondria forms an efficient Ca^2+^ transport channel, allowing Ca^2+^ to be transported from the ER to the mitochondria along the electrochemical gradient [38]. Chaperone glucose‐regulated protein 75 (GRP75) enhances the interaction between IP3R and VDAC1, thereby facilitating Ca^2+^ flow between the ER and mitochondria [39]. Several proteins enriched in MAMs are associated with the IP3R–GRP75–VDAC1 complex. For example, inositol‐requiring enzyme 1α (IRE1α) and Fun14 domain‐containing protein 1 (FUNDC1) act as scaffolding proteins at MAMs, physically interacting with IP3R to increase its abundance and stability at MAMs, thereby regulating Ca^2+^ flux between the ER and mitochondria [40, 41]. Given the importance of MAMs in the transfer of Ca^2+^ from the ER to the mitochondria, the length and thickness of tethering proteins may be critical factors affecting Ca^2+^ transport. Studies have shown that at an optimal distance (approximately 20 nm), ER and mitochondrial Ca^2+^ channels effectively interact and promote rapid Ca^2+^ transfer [40, 41]. Therefore, the binding of MAM‐tethering proteins, such as VAPB–PTPIP51, is essential for efficient mitochondrial Ca^2+^ uptake and may influence the interaction between IP3R and VDAC1 [42]. Under certain conditions, MAMs may also facilitate the reverse transfer of Ca^2+^ from the mitochondria to the ER. This has been demonstrated in studies where the ER Ca^2+^ pump, SERCA, was inhibited by TG, and considerable Ca^2+^ accumulation was still observed in the ER, possibly due to dynamic changes in the cellular demand for Ca^2+^ [130]. In summary, MAMs coordinate Ca^2+^ transport between the ER and mitochondria, which is crucial for maintaining cellular homeostasis.

### 3.2 Lipid Synthesis and Transport

Lipids play a vital role in regulating various life processes; serve as major components of cell and organelle membranes; and participate in energy metabolism, signal transduction, cell recognition, and material transport [131]. The ER and mitochondria are the primary hubs for eukaryotic membrane biogenesis and rely on lipid exchange at membrane contact sites. In addition to providing energy to the ER [132], mitochondria coordinate the synthesis of key membrane phospholipids, such as PE and phosphatidylcholine (PC) [133]. The biogenesis and respiratory functions of mitochondrial membranes are entirely dependent on lipids or lipid precursors provided by the ER [134]. The ER is capable of synthesizing nearly all membrane lipids, including phospholipids and cholesterol [135], and transporting them to the mitochondria through MAMs via nonvesicular transport [136].

Studies have shown that the protein kinase RNA‐like ER kinase (PERK) recruits ER–plasma membrane tethers and lipid transfer proteins such as E‐Syt1 at ER–mitochondria contact sites for mitochondrial lipid transport and respiration [48]. PC, PE, and CL are the main lipids constituting the mitochondrial membrane and account for 75–95% of the total lipids [137, 138]. PE is synthesized via three of four pathways in the ER [139], but for unknown reasons, almost no PE in mitochondria is derived from the ER [140]; instead, it is produced mainly from PS through the action of PS decarboxylase (PSD) in the inner mitochondrial membrane (IMM) [139]. The PS necessary for this process is synthesized by PS synthase (PSS) within the ER, and is subsequently transported to the mitochondria through lipid transfer proteins located on MAMs [43].

Multiple studies have indicated that proteins located in MAMs, such as oxysterol‐binding protein‐related proteins 5 and 8 (ORP5/8), MFN2, and CDP‐diacylglycerol synthase‐2 (CDS2), mediate the nonvesicular transport of PS from the ER to the mitochondria [44, 45, 46]. A small portion of PE produced in the mitochondria is sent back to the ER, where it is converted to PC through the CDP‐choline pathway or by the action of PE N‐methyltransferase (mainly expressed in the liver) [141].

Like PS, PC is produced in the ER and transported to the mitochondria through MAMs rather than being synthesized in the mitochondria [138]. CL biosynthesis occurs exclusively in the IMM and requires PA as a precursor, which is an input from the ER [142]. Studies have shown that the VAPB–PTPIP51 complex and lipocalin 2 play key roles in regulating the transport of PA from the ER to mitochondria through MAMs [33, 143]. Although cholesterol is present in low amounts in the mitochondria, it can be rapidly transported from the ER to other organelles. The ER, which lacks cholesterol, synthesizes cholesterol, which is quickly delivered to other organelles [144]. MAMs are rich in cholesterol, sphingolipids, and related enzymes, and form a membrane domain known as the intracellular lipid raft, which may create a local gradient conducive to the transport of cholesterol from the ER to the mitochondrial membranes [145]. The acute regulatory protein of steroidogenesis (StAR) associated with MAMs interacts with the ER chaperone, GRP78, to form a cholesterol complex at the MAMs, which is then transferred to the OMM and interacts with VDAC1/2, facilitating the transport of substrate cholesterol into the mitochondria for steroidogenesis [146]. However, excessive accumulation of cholesterol in the mitochondria can affect membrane fluidity and indirectly affect energy production efficiency. Knocking out molecules such as Caveolin‐1 and GRAMD1C may promote the transfer of cholesterol at mitochondrial–ER contact sites [49, 50], suggesting their role in regulating mitochondrial cholesterol metabolism.

In summary, MAMs are indispensable for lipid metabolism by promoting the transfer of lipids between the ER and mitochondria, regulating mitochondrial function, and affecting cellular metabolic pathways. The dynamic nature of MAMs and the participation of specific protein complexes highlight the importance of these structures in maintaining lipid homeostasis and overall cellular health.

### 3.3 Mitochondrial Homeostasis

MAMs regulate mitochondrial mass homeostasis. The regulation of mitochondrial mass homeostasis involves processes such as mitochondrial fusion, fission, and autophagy, which together maintain the number, structure, and function of mitochondria in a relatively stable, dynamic balance [147]. The core regulator of mitochondrial dynamics is the dynamin‐related GTPase family, which includes the fission‐promoting mitochondrial fission protein DRP1, the fusion‐promoting MFN1, MFN2, and optic atrophy 1 [148]. Mitochondrial fission occurs when the ER tubules surrounding the mitochondria contract before mitochondrial fission, marking the fission site [149]. DRP1 is recruited to the OMM, where it associates with its adaptors, mitochondrial fission factor (Mff), mitochondrial dynamics proteins of 49 kDa and 51 kDa (MiD49, MiD51), to form ring‐like or spiral structures around the site of mitochondrial constriction [51].

Several MAM proteins are involved in recruiting DRP1 to regulate fission processes, including syntaxin 17 (STX17) [54], disulfide bond A oxidoreductase‐like protein (DsbA‐L) [55], and phosphofurin acidic cluster sorting protein 2 (PACS‐2) [56]. Additionally, MAMs coordinate the licensing of mitochondrial DNA (mtDNA) replication with downstream mitochondrial fission events, distributing newly replicated mtDNA to daughter mitochondria [150]. DRP1 is not essential for mitochondrial fission; in the absence of DRP1, Fis1 can promote mitochondrial fission by inhibiting mitochondrial fusion [57]. Mitochondrial fusion results in mitochondrial elongation, a process controlled by MFN1 and MFN2 on OMM, and Opal on IMM [53]. MFN2 is sometimes located in the ER and physically interacts with MFN1 or MFN2 on OMM, where it participates in the connection between the ER and mitochondria [151]. MFN2 also interacts with other MAM components such as Sig‐1R, and promotes MFN2 oligomerization to regulate mitochondrial dynamics [52]. The tubular ER‐shaping protein, REEP5, interacts with MFN1/2 to regulate the “hitchhiking” behavior of mitochondria on the tubular ER, promoting the even distribution of mitochondria within the cell [58]. MFN2 is also involved in mitochondrial transfer mediated by mitochondrial–ER contact [152]. Intracellularly redundant or malfunctioning mitochondria are selectively eliminated through mitophagy, a highly conserved cellular process that maintains the equilibrium between mitochondrial quantity and the stability of cellular energy metabolism [153]. MAMs plays a key role in this process by regulating mitophagy via both ubiquitin‐dependent and nonubiquitin‐dependent pathways. PTEN‐induced putative kinase 1 (Pink1)/Parkin is the main protein in the ubiquitin‐dependent pathway. When mitochondria are damaged, Pink1 accumulates and phosphorylates Parkin, which ubiquitinates many OMM proteins, initiating mitophagy [154]. In the late stage of autophagy, BECN1 interacts with Pink1 in MAMs, promoting the formation of MAMs and autophagosomes [155]. FUNDC1 is a key protein in the nonubiquitin‐dependent pathway; under hypoxic conditions, Lon accumulates in MAMs and interacts with the FUNDC1–unc‐51‐like autophagy‐activating kinase 1 (ULK1) complex, inducing mitochondrial autophagy through chaperone activity [59]. In summary, MAMs regulate mitochondrial fission, fusion, and autophagy and participate in maintaining mitochondrial mass homeostasis.

### 3.4 ER Stress

ERS is an important defense mechanism by which cells respond to the accumulation of misfolded and unfolded proteins in the ER lumen and disturbances in Ca^2+^ balance to aid in the restoration of cellular homeostasis. To cope with ERS, the unfolded protein response (UPR) dynamically adjusts the folding and degradation capacity of the ER, restoring protein folding functions and reducing the concentration of unfolded proteins [156]. Moderate ERS can help restore normal ER function through UPR [157], whereas prolonged ERS can lead to cell death [158]. Under normal physiological conditions, PERK, IRE1α, and activating transcription factor 6 (ATF6) in the ER lumen are in an inactive monomeric state and are bound to BiP and GRP78. However, the accumulation of many unfolded proteins in the ER and their binding to BiP/GRP78 lead to the release of PERK, IRE1α, and ATF6α, initiating the UPR [159].

IRE1α accumulates in MAMs during ERS and splices XBP1 mRNA, upregulating the expression of folding enzymes and degradation enzymes and promoting the degradation of misfolded proteins [61]. The ERS sensor, ER Sig‐1R, associates with IRE1α in MAMs at the onset of ERS, allowing IRE1α to dimerize into a persistent, active nucleic acid endonuclease that enhances cell survival [60]. The mitochondrial ubiquitin ligase (MITOL) is enriched at the MAMs during ERS and interacts with IRE1α, which promotes the ubiquitination of IRE1α, thereby effectively suppressing the excessive activation of IRE1α‐induced ERS [62] Another ERS sensor, PERK, interacts with other proteins in the MAMs region, undergoes phosphorylation, and activates eukaryotic translation initiation factor 2α (eIF2α), inhibiting global protein synthesis, reducing the influx of nascent proteins into the ER, and selectively increasing the translation of some stress‐related proteins to alleviate ERS [63, 64]. Key proteins in MAMs, such as MFN2, act as upstream inhibitors of PERK activity and maintain ER homeostasis by directly interacting with and inhibiting PERK activation [65]. These findings indicate that proteins in MAMs play crucial roles in UPR signal transduction and the maintenance of cellular homeostasis.

### 3.5 Apoptosis

Apoptosis is a highly regulated programmed cell death process that is essential for maintaining homeostasis, promoting development, and defending against diseases in organisms [160]. Both mitochondria and ER are key organelles that mediate apoptosis [161], and the interaction between them is particularly important in the complex and finely regulated process of programmed cell death. The transfer of Ca^2+^ from the ER to mitochondria is crucial for maintaining mitochondrial function, but excessive accumulation of Ca^2+^ ions can induce mitochondrial Ca^2+^ overload, leading to the opening of the mitochondrial permeability transition pore (mPTP), mitochondrial swelling, membrane depolarization, rupture of the OMM, and release of cytochrome *c*, ultimately triggering apoptosis [162]. Cytochrome *c* released from the mitochondria into the cytosol is transferred to the ER, enhancing the function of IP3Rs and amplifying apoptotic signals [66].

IP3Rs play a central role in the regulation of mitochondrial–ER Ca^2+^ transfer and are regulated by multiple factors. For example, the serine–threonine kinase, Akt, preferentially phosphorylates IP3R3 in MAMs, inhibiting IP3R‐mediated ER–mitochondrial Ca^2+^ release and blocking apoptosis [67]. The BCL‐2 family of proteins also regulates IP3Rs. Specifically, Bcl‐xL can interact with IP3R, inhibiting Ca^2^⁺ release, thus affecting the initiation and progression of Ca^2^⁺‐dependent apoptosis [68, 69]. Another proapoptotic member of the Bcl‐2 family, Bax, also depends on MAMs for translocation to the mitochondrial membrane. Bax induces cytochrome *c* release and mediates apoptosis via mitochondrial Ca^2+^ accumulation and mPTP opening [70]. MAM‐associated proteins also play key roles in apoptosis. During apoptosis, the mitochondrial fission factor, Fis1, binds to Bap31, promotes ER–mitochondrial tethering, recruits and activates procaspase‐8, and initiates early apoptotic events [71]. Caspase‐8 further cleaves Bap31 into its proapoptotic form, stimulating ER Ca^2+^ release, leading to mitochondrial Ca^2+^ overload, and ultimately triggering apoptosis [72]. MAMs also appear to mediate apoptosis via the mitochondrial fission pathway. Studies have shown that BAX and DRP1 colocalize in the mitochondria during apoptosis, where they mediate mitochondrial permeabilization and fragmentation [73].

In summary, mitochondria–ER Ca^2+^ signaling, molecular platform regulation, and MAMs stability collectively form the core of the apoptotic signaling network, finely regulating the life and death decisions of cells.

### 3.6 Autophagy

Autophagy is a highly regulated, intracellular, self‐digestion process in which double‐membrane autophagosomes form and fuse with lysosomes to degrade cellular proteins, organelles, and other components, achieving material recycling and maintaining cellular homeostasis [67]. MAMs are key platforms for the formation of autophagosomes, bringing together various autophagy‐related proteins, such as autophagy‐related gene 14 (Atg14), Atg5, and BECN1, to regulate autophagosome formation and maturation [78]. In the early stages of autophagosome formation, starvation‐induced ULK1 translocates to MAMs, driving the assembly of the phosphatidylinositol 3‐kinase (PI3K) complex with Atg14 and BECN1, and initiating the generation of PI3P, thereby initiating autophagosome formation [74, 75]. Atg5, a marker protein for autophagosome formation, also translocates to MAMs where it elongates the autophagosome membrane and promotes transmembrane translocation of LC3 (microtubule‐associated protein 1 light chain 3) [76].

The elongation phase of autophagosomes involves the coordinated action of multiple key factors and processes. During the elongation phase of autophagosomes, Atg 2A (a subtype of Atg2) translocates to MAMs with the assistance of the TOM40 and TOM70 components of the mitochondrial translocase, where it interacts with Atg 9 to promote the growth of the phagophore and expand the volume of the autophagosome, thereby increasing autophagic flux [77]. ER‐resident STX17 interacts with Atg14 in MAMs, facilitating the final fusion of autophagosomes with lysosomes, whereas disruption of the mitochondria–ER contact site hinders the formation of Atg14 puncta [78].

Ca^2+^ transport between the ER and mitochondria is an important factor affecting autophagy. During autophagy, protein 2.4 induced by etoposide, translocates to MAMs and interacts with the core complex of IP3R–Grp75–VDAC, which helps maintain the integrity of MAMs structure and promotes autophagy [163]. When Ca^2+^ transport from the ER to mitochondria is inhibited, AMP‐activated protein kinase (AMPK), localized in MAMs, is activated by BECN1, promoting local autophagy [79]. Promyelocytic leukemia protein (PML) localizes to MAMs in a Ca^2+^‐dependent manner in P53‐driven cells and inhibits autophagy via the AMPK/mTOR/ULK1 pathway [80].

MFN2 is also involved in autophagy. Under energy stress, when mitochondrial fission occurs, a large quantity of AMPK translocates from the cytosolic solution to MAMs and interacts with MFN2, phosphorylating MFN2 and activating autophagy [81]. Autophagy also occurs in the absence of MFN2. For example, RTN3L, an effector of Rab32, promotes selective autophagy of proteins in MAMs [82].

In summary, autophagy is a complex process involving multiple proteins and organelles that work together and are precisely regulated. MAMs plays a pivotal role in this process by linking various autophagy‐related events.

### 3.7 Ferroptosis

Ferroptosis is a novel form of programmed cell death that is closely associated with abnormalities in iron metabolism and lipid peroxidation [164]. Ferroptosis is induced by lipid peroxidation in various subcellular membranes, including the ER, mitochondria, and lysosomes, with the ER being the key initial site of peroxidation [165]. In cells undergoing ferroptosis, the mitochondria often exhibit shrinkage, decreased size, increased membrane density, reduced cristae, degeneration or disappearance, and outer membrane rupture [166]. Therefore, communication between the ER and mitochondria may mediate ferroptosis. MAMs are involved in mediating intracellular iron homeostasis and that deletion of MAM components can activate an Aft1‐dependent iron deficiency response, leading to excessive iron accumulation within the cell [167]. Li et al. [83] reported that acute arsenic exposure induced the production of mitochondrial ROS (mtROS) in mouse lung epithelial cells, activated the PERK pathway, inhibited MFN2 expression, and ultimately led to MAMs dysfunction and ferroptosis, suggesting that mtROS‐induced MAMs dysfunction is at least partially related to arsenic‐induced ferroptosis and acute processes.

Similarly, increased MAMs formation was observed in ethanol‐exposed mouse liver cells with upregulated PERK expression and interaction with ACSL4, promoting lipid peroxidation and exacerbating ferroptosis [86]. In a recent study, the use of a fluorescent probe increased the number of MAMs during ferroptosis [168], further confirming the correlation between MAMs and ferroptosis. MAMs are also associated with cancer cell resistance to ferroptosis. The mechanisms by which MAMs regulate ferroptosis have been investigated. For example, tetrabromobisphenol A exposure‐induced ferroptosis in liver cells was found to upregulate the expression of GRP75, VDAC1, and MFN2, suggesting that Ca^2+^ between the ER and mitochondria may be involved in regulating ferroptosis [85]. This was confirmed in a study by Zhang et al. [169], who reported that defects in MAMs inhibited Ca^2+^ transfer between the ER and mitochondria, promoting the accumulation of DAG‐containing polyunsaturated fatty acids (PUFA) and increasing the resistance of cells to ferroptosis. These findings indicate that the resistance of cancer cells to ferroptosis is partly related to the Ca^2+^ transport function of MAMs. Under conditions of ferroptosis, the peripheral mitochondrial membrane protein A‐kinase anchoring protein 1 (AKAP1) anchors protein kinase A (PKA) in the mitochondrial MAMs, phosphorylating the S148 site of GRP75, causing GRP75 to be sequestered outside the mitochondria and interact with nuclear factor erythroid 2‐related factor 2 (Nrf2) and Kelch‐like ECH‐associated protein 1, leading to the transcriptional activation of a series of antiferroptosis genes [84]. Therefore, targeting MAMs may be a potential approach for promoting ferroptosis in cancer cells.

Zhuang et al. [170] discovered that the prodrug, LSA (LA‐SS‐ART, linoleic acid–disulfide–artemisinin), could promote MAMs formation and increase Ca^2+^ influx from the ER to the mitochondria by enhancing the interaction between IP3R and VDAC1, inhibiting mitochondrial β‐oxidation, triggering lipid peroxidation, simultaneously increasing the expression of Lpcat3 and MFN2, promoting phospholipid synthesis and increasing the production of PUFA–phospholipids, and synergistically enhancing ferroptosis [170]. In summary, MAMs play a key role in ferroptosis via calcium ion transport and lipid remodeling.

### 3.8 Inflammation

Inflammation is an essential mechanism by which the body combats injury and infection; however, chronic inflammatory responses can damage cells and tissues, leading to various diseases [171]. MAMs are necessary molecular platforms for formation of the NOD‐like receptor family pyrin domain‐containing 3 (NLRP3) inflammasome [172]. In the resting state, NLRP3 is located in the mitochondria and ER. When cells are subjected to stress, NLRP3 and its adaptor protein, apoptosis‐associated speck‐like protein, are relocalized to MAM components [87]. Possible reasons for the relocation of NLRP3 to MAMs include the release of DAMPs [173] or ROS [174] from mitochondria. ERS is also involved in mediating inflammation, and ERS‐induced activation of the NLRP3 inflammasome occurs through a Ca^2+^‐dependent and ROS‐independent mechanism associated with the upregulation of MAM‐resident chaperone proteins, increased mitochondria–ER contact, mitochondrial depolarization, and impaired dynamics [175].

The activation of the innate immune interferon gene stimulator (STING) also depends on MAM‐mediated Ca^2+^ signaling [176, 177]. Under high‐glucose conditions, the IP3R1‒GRP75‒VDAC1 axis mediates increased Ca^2+^ flow from the ER to the mitochondria, leading to mitochondrial Ca^2+^ overload, opening of the mPTP, release of mtDNA into the cytoplasm, binding to cyclic GMP‒AMP synthase (cGAS), and activation of STING [88]. The upregulation of STING also promotes its interaction with VDAC2, inhibiting MAMs formation mediated by VDAC2/GRP75, reducing mitochondrial Ca^2+^ uptake, and protecting mitochondrial function [89]. MAMs also enrich various inflammation‐responsive miRNAs including miR‐146a, miR‐142‐3p, and miR‐142‐5p [178]. In summary, MAMs play a central role in the cellular responses to inflammation and stress, which is crucial for understanding and treating diseases.

## 4 The Role of MAMs in Disease

**FIGURE 4:**
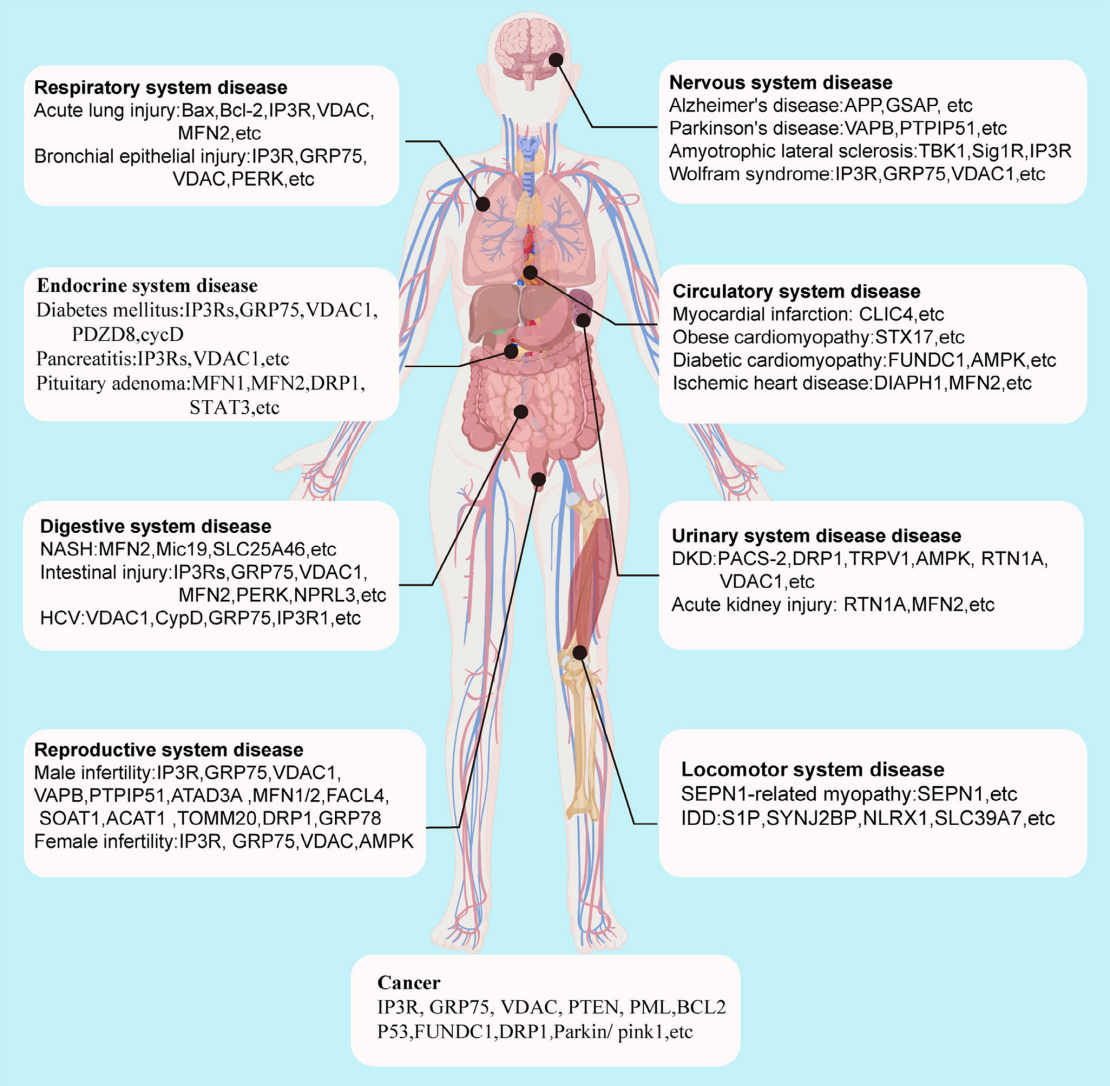
Role of MAMs in human diseases. Dysfunction of MAMs may serve as an initiating or exacerbating factor in the pathogenesis of various diseases. It plays a pivotal role in the pathophysiological mechanisms underlying common disorders and malignancies affecting the nervous system, circulatory system, urinary system, musculoskeletal system, respiratory system, endocrine system, digestive system, and reproductive system. Partial materials in this figure were created with BioGDP.com.

Given the critical role of MAMs in various cellular processes, such as calcium signaling, lipid metabolism, mitochondrial dynamics, ER homeostasis, and cell death, dysfunction of MAMs is closely linked to multiple pathological conditions. In this section, we summarize the roles of MAMs in common diseases and cancers across the eight major systems of the human body and the key proteins involved (Figure 4).

### 4.1 Role of MAMs in Nervous System Diseases

Neurological diseases are a group of disorders affecting the brain, spinal cord, and peripheral nerves, leading to dysfunction in the corresponding controlled areas and potentially other systemic conditions. MAMs influence not only the metabolism and secretion of lipids and proteins in neurons, calcium homeostasis, and energy production but also axonal transport and neurotransmission through interactions between the ER and mitochondria [179, 180]. Therefore, defects in MAMs levels may be the initial triggers of neurological diseases. Research on MAMs in neurological diseases is evolving rapidly, and MAMs dysfunction is associated with various neurological disorders such as Alzheimer's disease (AD), Parkinson's disease (PD), amyotrophic lateral sclerosis (ALS), and Wolfram syndrome. AD, one of the most common and earliest discovered neurodegenerative diseases associated with MAMs, is characterized by the accumulation of abnormally hyperphosphorylated Tau protein aggregates and extracellular amyloid‐beta (Aβ) plaques [181]. MAMs are enriched with amyloid precursor protein, presenilin‐1/2, and γ‐secretase‐activating protein, enzymes, or precursors involved in Aβ generation, and may influence the clearance rate of Aβ and Tau aggregates through autophagy regulation [182, 183]. Transcriptional modifications induced by inhibiting or knocking down class I histone deacetylases have potential therapeutic effects on improving mitochondrial–ER crosstalk in AD models [184].

An increase in Aβ aggregates can lead to a decrease in the mRNA levels of MAM proteins and a reduction in MAM length, causing intracellular calcium imbalance [184]. The binding of hyperforin to Aβ and its interaction with RyRs can regulate ER–mitochondria calcium levels and related signaling cascades, reducing Aβ‐induced neuronal apoptosis [185]. MAMs also play an important role in the pathogenesis of PD, with studies finding loose, shortened, and reduced MAM tethers in PD mouse models [186]. A proteomic analysis of MAMs in the midbrain identified 158 differentially expressed proteins [186]. Additionally, α‐synuclein, a marker of PD, can localize to MAMs and interact with VAPB, reducing its binding to PTPIP51 and ATP production [187]. MAMs disruption is a common pathological mechanism in ALS, potentially leading to the inactivation of TANK‐binding kinase 1 and exacerbating protein homeostasis stress [188]. Sig‐1R deficiency causes the dissociation of MAM components and mislocalization of IP3R, leading to calcium homeostasis imbalance in MAMs, activation of calpain, mitochondrial dysfunction, possibly causing ALS [189].

The primary defect in Wolfram syndrome involves poor ER calcium handling, which may be related to reduced Ca^2+^ transfer from the ER to the mitochondria through IP3R–GRP75–VDAC1 and decreased ATP production due to WFS1 or CISD2 deficiency [128]. These findings indicate that MAMs play crucial roles in various neurological diseases by regulating calcium signaling, energy metabolism, and other mechanisms that influence neuronal function and survival. A deeper understanding of these mechanisms will aid in the development of new therapeutic strategies to improve the prognosis of neurological diseases.

### 4.2 Roles of MAMs in Digestive System Diseases

The liver is the most studied organ of the digestive system in the field of MAMs. The liver plays a pivotal role in the digestive system, serves as a crucial metabolic organ, and performs functions, such as detoxification, bile secretion, and nutrient storage [190]. Additionally, the liver acts as a central regulator of lipid metabolism, primarily by coordinating the uptake of circulating lipids, de novo lipogenesis, fatty acid oxidation, and lipid export in very low density lipoprotein particles, to maintain a balance between lipid acquisition and disposal [191]. Consequently, lipid metabolism disorders caused by impaired MAM function are an important cause of liver disease. For example, a deficiency in liver MFN2 results in reduced PS transfer and phospholipid synthesis, leading to ERS and the development of alcoholic steatohepatitis (NASH)‐like phenotypes and hepatocellular carcinoma [45]. Similarly, the depletion of Mic19 in mice leads to decreased mitochondria–ER contact, disrupted mitochondrial lipid metabolism, and crista disintegration, damaging mitochondrial fatty acid β‐oxidation and lipid metabolism and potentially triggering NASH and liver fibrosis in mice [192].

Dysregulation of MAM‐mediated calcium regulation can also result in digestive system diseases. For example, CyD knockout disrupts MAMs integrity and interrupts IP3R‐mediated mitochondrial–ER calcium transport, ultimately leading to hepatic insulin resistance [193]. A similar situation occurs in intestinal diseases, in which enhanced IP3R‒MCU calcium axis‐mediated excessive calcium transfer in MAMs induces intestinal barrier damage, mitochondrial calcium overload, and dysfunction, which is one of the mechanisms by which deoxynivalenol damages the jejunum of piglets [194].

MAMs also mediate digestive system inflammation. Methane saline has been shown to inhibit the activation of MAMs and the NLRP3 inflammasome through the regulation of the PERK signaling pathway, thereby improving intestinal ischemia–reperfusion injury [195]. Furthermore, MAMs play an important role in viral replication. For example, hepatitis C viral proteins are specifically associated with MAMs in hepatitis C, although this association does not affect calcium signaling or glucose homeostasis between the ER and mitochondria [188]. These studies highlight the critical role of MAMs in digestive system diseases.

### 4.3 Roles of MAMs in Urinary System Diseases

MAMs have become a focal point of research in urinary system diseases, particularly kidney diseases. Wang et al. reported that vanadium exposure in duck kidneys resulted in alterations in 412 MAM proteins, indicating a high level of MAMs involvement in renal injury processes [21]. In a mouse model of diabetic kidney disease (DKD), high glucose levels increased the expression of mitogen‐activated protein kinase 1, which, in turn, reduced the levels of PACS‐2. This induced MAMs disruption and mitochondrial fragmentation. However, increasing PACS‐2 levels in HK‐2 cells ameliorated this effect [196]. Overexpression of PACS‐2 in HK‐2 cells prevents mitochondrial recruitment of DRP1, thus alleviating excessive mitochondrial division caused by high glucose concentrations; this subsequently restores MAMs integrity and enhances mitochondrial autophagy [56].

Excessive Ca^2+^ uptake by podocytes owing to mitochondrial dysfunction is a key feature of DKD. TRPV1‐mediated transient Ca^2+^ influx is reported to AMPK, thereby inhibiting the transcription of Fundc1. This reduces MAMs formation and Ca^2+^ transport from the ER to the mitochondria, a mechanism by which dietary capsaicin improves DKD [197]. Reticulon‐1A (RTN1A) expression is increased in human patients with DKD and RTN1A interacts with VDAC1, leading to the detachment of hexokinase‐1 from VDAC1. This triggers apoptosis and activation of the inflammatory pathway, contributing to tubular epithelial cell injury and loss [198]. Moreover, RTN1A‐mediated ERS may be a crucial determinant of the severity and maladaptive repair of acute kidney injury (AKI) [199]. These findings suggest that MAMs have promising research prospects in the field of urinary system diseases.

### 4.4 Roles of MAMs in Reproductive System Diseases

The reproductive system is an organ system tasked with the production of gametes (spermatozoa and ova) and their transport, fertilization, and the sustenance of nascent life [200]. Proteomic analysis of testicular MAMs in humans and mice indicated the highly conserved expression of 1,347 proteins specific to MAMs in the testes [201]. These testis‐specific MAM proteins have been implicated in spermatogenesis, male gamete production, and sexual reproduction. Latino et al. [202] demonstrated that d‐aspartate enhanced MAMs function by increasing lipid transfer from the ER to the mitochondria and calcium signaling, while reducing ERS, which is essential for efficient steroidogenesis in the testes. Disruption of MAMs function can lead to reproductive system diseases. For example, exposure of female mice to di‐(2‐ethylhexyl) phthalate increased the risk of ERS in atretic follicles, disturbed intracellular calcium homeostasis in oocytes, altered the structure and function of MAMs, and ultimately induced apoptosis [203]. These findings suggest that targeting MAMs could be a potential therapeutic strategy for the treatment of reproductive system diseases. For example, the chlorpromazine derivative, JX57, has shown promising effects in the treatment of endometrial cancer, possibly by targeting GRP75. Mechanistically, JX57 directly binds to GRP75, inhibiting the formation of the IP3R‒GRP75 complex, damaging the structure of MAMs and disrupting calcium homeostasis between the ER and mitochondria, leading to a mitochondrial energy crisis and AMPK activation [204].

Overall, MAMs are crucial for normal reproductive system function and is closely associated with the development of various reproductive system diseases. Targeting MAMs are considered a potential therapeutic strategy, although research is still in its early stages and further exploration is needed to advance related treatments.

### 4.5 Roles of MAMs in Circulatory System Diseases

Cardiovascular diseases, including heart diseases, are among the most prevalent circulatory system disorders. Cardiac myocytes contain chloride intracellular channel protein (CLIC4), which plays a role in regulating calcium homeostasis between the ER and mitochondria. The absence of CLIC4 is associated with an increased risk of myocardial infarction [205]. In obese cardiomyopathy, elevated STX17 levels promote MAMs formation, leading to mitochondrial Ca^2+^ overload, accumulation of mitochondrial O^2−^, lipid peroxidation, and subsequent cardiac damage [206]. Research on diabetic cardiomyopathy has indicated that high glucose levels inhibit AMPK, promoting MAMs formation mediated by Fundc1 and excessive mitochondrial fission, resulting in myocardial injury [11].

MAMs are also implicated in angiogenesis; for example, TMEM215 was found to protect endothelial cells from BIK‐induced mitochondrial apoptosis through the downregulation of EZH2 in response to shear stress derived from blood flow [207]. Additionally, the cytoplasmic F‐actin‐polymerizing protein, DIAPH1, facilitates ischemia–reperfusion‐induced cardiac injury by increasing the proximity of mitochondria to the ER through interaction with MFN2 [208]. FUNDC1‐mediated MAMs contribute to cardiac protection against DIC by restoring the biosynthesis of obstructed autophagosomes [209].

In summary, studies on circulatory system diseases have indicated the mechanisms of action of MAMs and other factors, thereby providing potential directions for disease treatment.

### 4.6 Roles of MAMs in Endocrine System Diseases

The endocrine system is a complex network composed of glands and tissues in the human body that secretes hormones into the blood to regulate various physiological functions. Diabetes mellitus (DM), one of the most common endocrine disorders [210] and is characterized by hyperglycemia, with manifestations of insulin secretion defects and insulin resistance [211]. Pancreatic β‐cells rely on Ca^2^⁺ signals to trigger the release of insulin granules. Overexpression of IP3Rs may lead to the abnormal uptake of mitochondrial Ca^2^⁺, resulting in abnormal morphology and function of the mitochondria, thus causing a reduction in insulin secretion or a disordered secretion pattern [212].

MAMs are also involved in mediating pancreatic β‐cell death. Tiwary et al. [213] reported that in palmitate‐induced pancreatic β‐cell apoptosis, the overexpression of GRP75 increased the physical coupling between the ER and mitochondria, promoting the transfer of Ca^2^⁺ to mitochondria, which is related to increases in the mitochondrial membrane potential and ROS. Similarly, PDZD8 is highly expressed in DM and regulates mitochondria–ER contact and calcium dynamics through enhanced interactions with cyclophilin D and IP3R‒GRP75‒VDAC, inducing pancreatic β‐cell death [96]. Therefore, targeting the calcium channels in MAMs may offer therapeutic potential for the treatment of endocrine disorders.

Urolithin A has been shown to protect against severe acute pancreatitis by modulating ER–mitochondrial calcium channels and reducing acinar cell necroptosis [214]. MAMs also participate in gluconeogenesis. The lncRNA H19 can suppress the impairment of mitochondria–ER junctions in hepatocytes and potentiate gluconeogenesis by regulating the expression level of VDAC1 [215]. Collectively, these studies suggest that MAMs hold great promise as therapeutic targets for endocrine system‐related diseases.

### 4.7 Roles of MAMs in Locomotor System Diseases

The human locomotor system, namely, the musculoskeletal system, consists of bones, skeletal muscles, ligaments, tendons, joints, cartilage, and other connective tissues and plays crucial roles in locomotion, support, and protection [216]. Abnormalities in MAMs are closely linked to skeletal muscle dysfunction. For example, SEPN1‐related myopathy caused by SEPN1 mutations is characterized by muscle weakness and fatigue, leading to scoliosis and potentially life‐threatening respiratory failure [217]. SEPN1 is highly concentrated in MAMs, and its deficiency results in reduced mitochondria–ER connections, decreased intracellular calcium concentrations in organelles, and impaired oxidative phosphorylation [218]. Furthermore, BCL2L13, a member of the BCL‐2 family, is highly expressed in endurance‐trained individuals and localized to MAMs. BCL2L13 interacts with calcium ion signaling channels, such as VDAC1, potentially playing an important role in maintaining calcium homeostasis and skeletal muscle function [219].

MAMs are vital for maintaining normal spinal function. Sphingosine‐1‐phosphate depletion can lead to intervertebral disc degeneration (IDD) through vesicular transport, mitochondria–ER calcium flux, and oxidative stress pathways [220]. SYNJ2BP, located in nucleus pulposus (NP) cells, maintains MAM connections and the formation of the NLRX1–SLC39A7 complex, aiding in the maintenance of zinc ion (Zn^2^⁺) homeostasis within mitochondria. The loss of SYNJ2BP exacerbates MAMs damage and inhibits the formation of the NLRX1–SLC39A7 complex, potentially reducing the localization of SLC39A7 in the mitochondria, increasing mitochondrial zinc ion accumulation, and ultimately leading to the exacerbation of mitochondrial dysfunction, accelerated aging of NP cells, and progression of IDD [221].

Overall, the structure of MAMs is essential for proper muscle and bone function. Research on molecules such as SEPN1 and BCL2L13 has indicated new mechanisms underlying related diseases, thus offering new avenues for therapeutic strategies.

### 4.8 Role of MAMs in respiratory System Diseases

The respiratory system consists of the airways and lungs, the latter being the primary organ responsible for the inhalation of oxygen and exhaled carbon dioxide, thereby maintaining normal physiological functions in the body [222]. The role of MAMs in respiratory diseases is being increasingly recognized. MAMs dysfunction is a key factor in lung injury. For example, Xiao et al. showed that paraquat was toxic to rat lung tissue cells. Paraquat can induce ER Ca^2^⁺ overload, and the activated immunoglobulin binding protein (BIP) and transcription factor C/EBP‐homologous protein (CHOP) pathways can directly or indirectly affect the expression of apoptosis‐related caspase family factors [223]. Moreover, paraquat promoted ER Ca^2^⁺ release and induced mitochondrial Ca^2^⁺ uptake. This process triggers a response of Bax/Bcl‐2 channel proteins to the IP3R/VDAC/MCU calcium axis, resulting in the disruption of this axis and cytochrome *c* release, ultimately leading to ERS and cell apoptosis [223]. In addition, arsenic‐induced pulmonary ferroptosis and acute lung injury have been proven to be, at least partially, related to MAMs dysfunction triggered by mtROS [83]. MAMs dysfunction is also associated with bronchial disease. Titanium dioxide nanoparticles can induce ERS in human bronchial epithelial cells and disrupt the MAMs and Ca^2^⁺ balance, thereby increasing autophagy [224]. These findings illustrate the complex relationship between MAMs dysfunction and various respiratory diseases.

### 4.9 Role of MAMs in Cancer

Research on the involvement of MAMs in different types of cancer and their roles in tumor growth and metastasis is a field of exponential growth. Owing to their rapid proliferation and invasive nature, cancer cells require various methods to meet their energy and material demands, such as increasing glucose uptake and glycolytic activity (known as the Warburg effect), lipid synthesis and degradation, and modulation of Ca^2+^ signaling [225]. Therefore, communication between ER and mitochondria plays a crucial role in cancer initiation and progression. Aberrant expression of MAM proteins is commonly observed in tumors, where changes in the cellular microenvironment are often associated with the activation of transmembrane receptors and Ca^2+^‐permeable channels involved in cancer progression, epithelial–mesenchymal transition, invasion, and resistance to apoptosis [226].

The IP3R–GRP75–VDAC–MCU complex, which plays a central role in MAMs Ca^2+^ transport, is regulated by oncogenes, such as *PTEN*, *PML*, and *BCL2*. In MAMs, PTEN binds to IP3R and prevents its degradation, thus promoting Ca^2+^ transport into mitochondria, which is crucial for apoptosis [227]. Loss of PTEN function and subsequent inappropriate Ca^2+^ transport can lead to resistance to apoptosis [228, 229]. Similarly, when PML is directed to MAMs by p53, it affects Ca^2+^ transfer from the ER to the mitochondria, thereby regulating apoptosis and autophagy. Therefore, its absence can promote tumor development by providing a growth advantage to tumor cells that use autophagy as a survival strategy under stress conditions [80]. Conversely, *BCL2*, another oncogene enriched in MAMs, directly interacts with IP3R, preventing Ca^2+^ transport from the ER to the mitochondria and interfering with the release of cytochrome *c* from the mitochondria, thus playing a role in resisting apoptosis in cancer [230]. High IP3R expression is associated with increased invasiveness and metastasis of cancer cells [231].

MAMs also influence tumor angiogenesis by modulating Ca^2+^ transfer [207]. For example, increased MAMs formation in cancer cells leads to elevated cytosolic Ca^2+^ levels, promoting the phosphorylation of serum response factor (SRF) and enhancing the binding of SRF to the VEGFR2 promoter, resulting in increased VEGFR2 production and subsequent angiogenesis. Silencing of FUNDC1 reduces MAMs formation and inhibits tumor angiogenesis [232]. In breast cancer, the extracellular matrix enhances ER‐mediated Ca^2+^ transport to the mitochondria, promoting DRP1 recruitment to the mitochondria and phosphorylation at serine 616, and inducing mitochondrial fragmentation and Parkin/PINK1‐mediated mitophagy, which is beneficial for tumor cell survival [233]. Furthermore, cancer cells often exhibit alterations in inner membrane CL and cholesterol levels, which may confer resistance to apoptosis or metabolic reprogramming [234]. The tumor suppressor, p53, regulates PA transport to the mitochondria through MAMs [235]. In drug‐resistant cells, the transfer of Ca^2+^ from MAMs to the mitochondria is not essential for reducing apoptosis; however, disrupting sphingolipid biosynthesis in MAMs and lipid transfer to the OMM can decrease apoptotic signaling and enhance multidrug resistance in recurrent cells [236]. These studies highlight the diverse roles of MAMs in cancer.

## 5 Potential Therapeutic Targets

Given the multifaceted roles of MAMs in disease processes, targeting MAMs has emerged as a potential therapeutic strategy [237]. As critical functional contact sites between the ER and mitochondria, MAMs coordinate essential cellular processes including intracellular signaling, lipid metabolism, and Ca^2^⁺ homeostasis. Precise modulation of MAMs functionality offers novel perspectives for developing interventions across multiple disease states (Table 2).

**TABLE 2:** Therapeutic strategies targeting MAMs in disease prevention and intervention.

Therapeutic strategy	Specific measures	Representative drug/target	Specific mechanism	Disease	References
Pharmacological intervention	Ca^2+^ signaling modulation	CGI1746	Blocks Ca^2+^ transfer from the endoplasmic reticulum to mitochondria and promotes the accumulation of triacylglycerols rich in PUFAs, thereby inhibiting ferroptosis	Acute kidney injury	[169]
		Ursolic Acid	Inhibits the AhR‐mediated expression of TGM2, reducing mitochondrial Ca^2+^ influx	Diabetes mellitus	[238]
		Xestospongins	Inhibits IP3R‐mediated Ca^2+^ overload	Cardiovascular diseases	[239]
		LSA	Promotes MAMs formation and mitochondrial Ca^2^⁺ influx, facilitating ferroptosis	Cancer	[170]
		Empagliflozin	Inhibits SGLT2, activating the AMPK pathway, decreasing podocyte MAMs formation	Diabetic kidney disease	[240]
	Mitochondrial dynamics regulation	Mdivi‐1	Inhibits DRP1 to prevent excessive mitochondrial fission and maintain the structural integrity of MAMs	Huntington's disease	[241]
	Lipid metabolism modulation	Mitotane	Inhibition of SOAT1 triggers lipid‐induced ER stress and apoptosis​	Adrenocortical carcinoma	[242]
Gene editing​	RNAi	IP3R	Inhibits IP3R‐dependent activation of the mPTP	Immune kidney injury	[243]
	CRISPR/Cas9	PERMIT	Knockout of PERMIT blocks the transport of CerS1 to the mitochondrial membrane, thereby alleviating ceramide‐dependent mitophagy	Cancer and/or neurodegeneration	[244]
	AAV9	ARL6IP1	Restore the level of ARL6IP1 and reverse the pathological manifestations	Hereditary spastic paraplegia	[245]
Lifestyle Interventions​	Moderate exercise	Moderate‐to‐high‐intensity exercise	Promotes MAMs formation in the liver of HFD mice, thereby alleviating hepatic insulin resistance	Diabetes mellitus	[246]
	Dietary adjustments	ω‐3 Fatty acids	Regulates the structure and function of MAMs, affecting Ca^2+^ homeostasis, mitochondrial function, inflammation, and antioxidant activity, thereby exerting protective effects against insulin resistance	Diabetes mellitus	[247]

In normal tissues, therapeutic strategies focusing on key MAM‐associated proteins demonstrate considerable translational potential. Ca^2+^ signaling modulators represent a primary approach, where inhibition of excessive Ca^2^⁺ transfer (via calcium channel blockers) or enhancement of calcium buffering capacity (through calcium‐binding protein activators) can mitigate mitochondrial Ca^2^⁺ overload‐induced cellular damage. For example, the small molecule, CGI1746, targets the MAM chaperone, sig‐1R, to block the transfer of Ca^2+^ from the ER to the mitochondria and promote the accumulation of PUFA‐containing triacylglyerols, thereby inhibiting ferroptosis and protecting mice from AKI [169]. Similarly, urolithin A has been shown to inhibit AhR‐mediated TGM2 expression in murine models by disrupting MAMs, inhibiting mitochondrial Ca^2+^ influx, and reducing mtROS accumulation and amyloid protein production in neurons under high glucose conditions [238]. In neurodegenerative contexts, Xestospongins exemplify therapeutic candidates that preserve mitochondrial function by inhibiting IP3R‐mediated Ca^2+^ overload through MAM pathways [239]. Mitochondrial dynamics regulators present another therapeutic avenue, as exemplified by the DRP1 inhibitor, Mdivi‐1, which maintains MAMs structural integrity by preventing excessive mitochondrial fission, thereby ameliorating energy metabolism dysfunction in ischemia–reperfusion injury [241]. Metabolic modulators like the SGLT2 inhibitor, empagliflozin, which is widely used in patients with diabetes, reduces podocyte MAMs formation in mice by activating the AMPK pathway, thereby alleviating kidney injury [240]. These findings collectively emphasize the therapeutic value of modulating MAMs functional states for disease prevention and intervention.

In malignant cells, MAMs manipulation represents a dual strategy to induce tumor cell death and suppress metastasis. The prodrug, LSA, exemplifies this approach by enhancing MAMs formation to amplify ER‐to‐mitochondria Ca^2^⁺ flux, thereby triggering ferroptosis in chemoresistant cancer cells [170]. Disruption of MAM‐mediated lipid transport constitutes another key mechanism, as demonstrated by mitotane, the only approved chemotherapeutic agent for the treatment of adrenocortical carcinoma (ACC) [248], which induces the accumulation of toxic lipids, including free and oxidized cholesterol, in ACC cells by inhibiting sterol O‐acyltransferase 1 (SOAT1), thereby triggering lipotoxic ERS [242]. SOAT1 is enriched in MAMs and protects cells from the harmful effects of free cholesterol by converting it to cholesterol esters [249]. Targeting MAM proteins to overcome chemoresistance caused by repeated chemotherapy drug use is another potential strategy. For example, Koczian et al. [250] discovered that in leukemic cells, the protein disulfide isomerase inhibitor, PS89, directly affected PDI and BAP31, amplifying ER and mitochondrial stress triggers, resulting in a potent chemosensitizing effect.

Emerging genetic technologies further expand therapeutic possibilities. Targeting MAM regulatory factors, gene silencing techniques such as RNA interference (RNAi) or CRISPR/Cas9 can be used to reduce the progression of MAM‐related diseases by silencing specific genes or to enhance MAMs function or compensate for gene deficiencies by introducing specific genes using viral or nonviral vectors. RNAi effectively suppresses IP3R‐dependent mPTP activation by preventing Ca^2+^ overload [243], whereas CRISPR/Cas9‐mediated PERMIT ablation blocks ceramide synthase 1 (CerS1) trafficking to mitochondrial membranes, thereby attenuating ceramide‐dependent mitophagy [244]. Genome‐wide CRISPR screening has identified GET4 deletion as a neuroprotective modulator through enhanced ER–mitochondria tethering [251]. Gene therapy applications demonstrate therapeutic potential in hereditary spastic paraplegia (HSP) models, where ARL6IP1 restoration rescues ER–mitochondria homeostasis through LC3B/BCl2L13 interactions, ameliorating HSP‐associated pathophysiology [245].

Lifestyle interventions, including moderate aerobic exercise and dietary adjustments, have emerged as viable approaches to modulate the function of MAMs. The regulation of MAMs through a balanced dietary structure has been shown to have a substantial impact. It is well established that abnormalities in MAMs function, induced by high‐fat diets, are critical factors in exacerbating insulin resistance and promoting apoptosis [252, 253]. Furthermore, ketogenic diets, which reduce MAMs numbers and inhibit mitochondrial function, have been implicated in suppressing regulatory T cells and promoting cardiac fibrosis, thereby adversely affecting ventricular function and cardiac remodeling in the progression of diabetic cardiomyopathy [254]. Consequently, a low‐fat, healthy diet, particularly one rich in PUFA, is believed to influence MAMs function by modulating lipid metabolism and calcium homeostasis. For instance, ω‐3 fatty acids have been demonstrated to exert protective effects against insulin resistance by regulating the structure and function of MAMs, influencing calcium homeostasis, mitochondrial function, inflammatory responses, and antioxidant activities [255]. Additionally, exercise training is considered one of the effective stimuli for improving insulin resistance [247]. Studies have shown that moderate to high‐intensity exercise can effectively promote the formation of MAMs in the livers of high‐fat diet‐fed mice, thereby alleviating hepatic insulin resistance [246]. However, the specific effects and safety of these interventions require further clinical trials and research to validate. Although most studies are currently at the laboratory stage, they have provided potential therapeutic strategies for future treatments of diseases associated with MAMs dysfunction.

Advances in MAMs biology have determined novel pathogenic mechanisms and identified multitiered therapeutic targets encompassing pharmacological agents, gene‐editing tools, and metabolic interventions. Although tool compounds like Xestospongins and Mdivi‐1 have elucidated specific pathways, challenges remain in tissue‐specific delivery and off‐target effects. Although CRISPR technologies enable precise genomic editing, their long‐term safety and regulatory stability require rigorous evaluation [256, 257]. Current preclinical investigations face critical translational gaps, particularly regarding the druggability of MAMs proteins using  Food and Drug Administration (FDA)‐approved agents. Future research should integrate multiomics approaches to delineate MAMs regulatory networks and explore synergistic effects between pharmacological and lifestyle interventions, ultimately accelerating the translation of MAMs‐targeted therapies into clinical practice.

## 6 Outlook

MAMs are highly specialized structural domains with intricate architecture and complex functions in organellar membranes. MAMs play a crucial role in human health and disease by mediating communication and interactions between the ER and mitochondria. For example, Morcillo et al. [258] reported that the function of MAMs was considerable disrupted in every disease they investigated, underscoring their universal relevance as cross‐disease therapeutic targets. Although this broad association provides a unique lens for deciphering disease mechanisms, it also underscores the necessity for interventions that commensurately modulate multiple pathways.

The therapeutic potential of MAMs arises from their dynamic role in maintaining cellular homeostasis. Their assembly and disassembly are precisely regulated by tethering proteins such as MFN2, PACS‐2, and VAPB–PTPIP51, forming a molecular basis for developing small‐molecule drugs or gene‐editing tools (e.g., CRISPR/dCas9) [181, 259, 260, 261]. Selective modulation of specific interaction nodes can restore MAMs equilibrium under pathological conditions while circumventing systemic ER–mitochondria connectivity disruption, enabling precision therapy. Disease‐specific strategies have emerged from this dynamic regulatory framework: suppressing excessive MAMs connectivity in neurodegenerative disorders alleviates mitochondrial Ca^2^⁺ dyshomeostasis and neuronal degeneration [262], whereas targeting MAMs‐mediated Ca^2^⁺ signaling in cancer counteracts chemoresistance and enhances drug sensitivity [225]. Advances in nanodelivery systems (e.g., liposomes or exosome‐based carriers) now permit targeted intervention in MAMs microdomains by overcoming organelle membrane barriers [263], while CRISPR/Cas9‐mediated gene editing enables precise genetic regulation for personalized therapies [261]. Integrating these approaches—dynamic interaction control, disease pathway analysis, and nanotechnology—promises to transcend the limitations of conventional therapies in modulating organelle networks, offering transformative solutions for disease management.

Technological innovation has propelled MAMs research into a new era (Table 3). Super‐resolution microscopy, molecular dynamic tracking, and multiomics analysis now form the cornerstone of this field. Stimulated emission depletion (STED) microscopy bypasses diffraction limits to resolve MAMs membrane protein distributions and nanoscale structural domains [272, 273], whereas single‐molecule localization microscopy (SMLM) achieves nanometer‐scale precision in mapping dynamic protein behaviors [274]. Cryo‐electron tomography (cryo‐ET) further quantifies subnanometer ultrastructural changes at single‐cell resolution [264]. Fluorescence resonance energy transfer (FRET) and fluorescence lifetime imaging microscopy (FLIM) monitor real‐time protein interactions and microenvironmental fluctuations within MAMs [265, 266], complemented by spatiotemporal proximity labeling techniques (Split‐TurboID) that capture interactome dynamics in live cells [275, 276]. The recently developed orthogonal dual‐labeling technique (OrthoID)—using TurboID and APEX2 to biotinylate and adamantylate proteins proximal to OMM and ERM, respectively, substantially reduces false positives from endogenous biotinylated proteins, enabling the identification of novel MAMs‐associated proteins [267]. Additionally, artificial intelligence (AI)‐driven integration of multimodal data with live imaging is overcoming historical bottlenecks in dynamic tracking and cross‐scale analysis [268, 269, 270]. For instance, microfluidics‐coupled super‐resolution microscopy combined with Moore–Neighbor tracking algorithms achieves single‐molecule localization within narrow membrane contact zones (<20 nm), refining the minimal detectable MAMs distance to ∼8 nm [122]. These technological leaps are transforming MAMs research from static observation to dynamic precision control, providing multidimensional insights into their functional mechanisms.

**TABLE 3:** Advanced technologies for investigating and targeting MAMs.

Technology category	Technology name	Description	Application examples	References
Genetic technologies	RNAi	Silences specific genes to mitigate MAM‐associated disease progression	RNAi inhibits IP3R‐dependent mitochondrial permeability transition pore (mPTP) activation, preventing Ca^2^⁺ overload.	[243]
	CRISPR/Cas9	Enables precise gene knockout or repair of MAM‐related genes	PERMIT ablation blocks CerS1 transport to mitochondrial membranes, reducing ceramide‐dependent mitophagy.	[244]
Imaging and detection technologies	Super‐resolution microscopy (STED, SMLM, Cryo‐ET)	Breaks optical diffraction limits to resolve nanoscale MAMs structures	Cryo‐ET quantifies subnanoscale ultrastructural changes in MAMs	[264]
	Fluorescence techniques (FRET, FLIM)	Real‐time monitoring of protein interactions and microenvironmental changes within MAMs	FRET and FLIM track protein–protein interactions and Ca^2^⁺ transients in MAMs.	[265, 266]
	Proximity labeling (Split‐TurboID, OrthoID)	Tags proximal proteins in live cells to capture dynamic MAMs interactomes	OrthoID combines TurboID and APEX2 for reduced false positives, identifying novel MAM‐associated proteins.	[267]
Nanotechnology	Nanocarrier systems (liposomes, exosomes)	Facilitates targeted drug delivery across organelle membranes to MAMs microdomains	Liposomal or exosomal carriers enable MAM‐specific interventions, enhancing therapeutic efficacy.	[263]
Computational & AI technologies	AI‐driven multiomics integration	Combines dynamic imaging with multiomics data to overcome tracking and cross‐scale analysis challenges	AI integrates super‐resolution microscopy, microfluidics, and metabolic monitoring to predict key regulatory nodes.	[268, 269, 270]
Multiomics & modeling technologies	Multiomics profiling (proteomics, lipidomics, metabolomics)	Systematically analyzes MAMs regulatory networks and metabolic correlations	Quantitative subcellular reconstruction reveals a lipid mediated interorganelle biogenesis network.	[271]
	Organoid & genome‐edited animal models	Simulates disease‐specific MAMs dysfunctions to validate therapeutic strategies	Patient‐derived iPSC organoids and genome‐edited animal models study MAMs roles in neurodegenerative diseases.	[261]

However, the therapeutic targeting of MAMs continues to present formidable multidimensional challenges that span fundamental biological understanding to clinical translation due to their intricate structural organization and dynamic functional plasticity. MAMs exhibit remarkable tissue‐specific heterogeneity at the molecular level, as demonstrated by comprehensive proteomic profiling studies identifying over 1000 and 4000 distinct MAM‐associated proteins in skeletal muscle and renal tissues respectively, a finding that fundamentally constrains the development of universal MAM‐targeting pharmacological agents [21, 277]. This molecular diversity is further complicated by the context‐dependent functionality of core MAM regulatory proteins, which frequently demonstrate pleiotropic roles in mediating interorganelle communication networks, often resulting in paradoxical outcomes when targeted therapeutically, a phenomenon starkly illustrated by contradictory reports regarding MFN2's dichotomous involvement in ferroptosis pathways. The inherent challenge of achieving precise spatial targeting is exacerbated by the nonexclusive subcellular localization of many MAM‐resident proteins, with interventions frequently producing off‐target consequences, as exemplified by mdivi‐1's dual functionality in simultaneously inhibiting DRP1‐mediated mitochondrial fission while adversely disrupting Ca^2^⁺ homeostasis and metabolic function in oligodendroglial cells [278]. A deeper layer of complexity arises from extensive functional redundancy among MAMs structural components and enzymatic effectors, particularly evident in critical processes such as phospholipid exchange where multiple proteins including ORP5/8, MFN2, and CDS2 participate in PS transport through mechanisms that remain incompletely characterized, with their potential compensatory relationships and competitive interactions posing considerable obstacles to rational drug design [44, 45, 46]. necessitating temporally precise and dosage‐controlled therapeutic strategies. The pathophysiological relevance of MAMs is further obscured by their nonlinear, disease‐stage‐dependent spatiotemporal dynamics, wherein decreased MAMs connectivity has been mechanistically linked to early metabolic dysfunction in insulin resistance through impaired lipid handling [253], whereas excessive MAMs formation in late‐stage diabetes triggers cell death [96], necessitating precise temporal and dosage control. Technically, conventional microscopy lacks resolution for nanoscale MAMs structures, whereas super‐resolution techniques (STED, cryo‐ET) face limitations in live imaging speed and sample preparation [279, 280]. The field suffers from a critical shortage of highly specific molecular probes capable of selectively interrogating MAMs components without perturbing their native functional states, whereas millisecond‐scale signaling events such as rapid Ca^2^⁺ transients continue to evade comprehensive characterization due to technical limitations in detection methodologies. Existing in vitro model systems frequently fail to adequately recapitulate the complex three‐dimensional architecture and biochemical microenvironment of native MAMs interfaces, and although emerging approaches incorporating organoid cultures and transgenic animal models show promise, they remain in relatively early stages of development and validation. Perhaps most importantly, the field currently lacks robust computational frameworks and standardized analytical pipelines for effectively integrating multidimensional omics datasets (including proteomic, lipidomic, and metabolomic profiles) with dynamic network modeling approaches, creating a critical bottleneck in deriving comprehensive, systems‐level understanding of MAMs regulatory mechanisms and their therapeutic potential.

To address these challenges, future research must prioritize four directions: first, developing noninvasive molecular probes and optogenetic tools for reversible, dynamic MAMs modulation. Second, integrating super‐resolution imaging, single‐cell spatial omics, and real‐time metabolic monitoring to construct three‐dimensional dynamic interaction models. Third, leveraging patient‐derived iPSC organoids and gene‐edited animal models to recapitulate disease‐specific MAMs dysfunction. Fourth, using AI‐driven computational biology to integrate multiomics data, predict critical network nodes, and screen targeted therapeutics. Through multidisciplinary collaboration and preclinical model optimization, MAMs research is poised to advance from mechanistic exploration to precision therapeutics, offering innovative strategies to combat neurodegenerative diseases, metabolic syndromes, and cancers.

## 7 Conclusion

As critical signaling and metabolic hubs bridging the ER and mitochondria, MAMs serve as central regulators of cellular homeostasis by orchestrating Ca^2^⁺ dynamics, lipid metabolism, mitochondrial quality control, and ER homeostasis [281, 282]. Accumulating evidence demonstrates that dysregulation of MAMs architecture and functionality is intimately linked to the pathogenesis of diverse disorders, including neurodegenerative, cardiovascular, and kidney diseases [283]. Although substantial progress has been made in elucidating MAM biology, fundamental challenges persist, including incomplete understanding of their intrinsic regulatory mechanisms, limitations in precise structural and functional characterization, and the pleiotropic effects of potential therapeutic targets that complicate drug development.

Future advances in super‐resolution imaging, multiomics profiling (proteomics, lipidomics, and metabolomics), and single‐cell technologies are expected to provide unprecedented insights into MAMs organization and dynamics. Systematic identification and validation of core MAM‐associated molecular signatures and signaling networks may indicate novel druggable targets, whereas emerging approaches such as CRISPR‐based gene editing and small‐molecule modulators could enable precise manipulation of MAMs function. Collectively, these developments hold considerable potential for translating MAMs research into targeted therapeutic strategies.

In summary, MAMs represent a pivotal interface for interorganelle communication, with profound implications in both physiological and pathological contexts. Deciphering the molecular mechanisms governing MAMs regulation and their disease‐associated perturbations will not only advance our fundamental understanding of cellular homeostasis but also open new avenues for the treatment of MAM‐related disorders.

## Author Contributions

Yong Liu, Zi‐Hui Mao, and Qi Feng conceptualized and wrote the manuscript. Yong Liu, Zi‐Hui Mao, Junwen Huang, Hui Wang, Xiao Zhang, Xin Zhou, Yue Xu, Shaokang Pan, Dongwei Liu, Zhangsuo Liu, and Qi Feng reviewed and revised the manuscript. Dongwei Liu, Zhangsuo Liu, and Qi Feng made substantial contributions to the design and critical revision of the manuscript. All authors have seen and approved the final version of the manuscript being submitted.

## Ethics Statement

The authors have nothing to report.

## Conflicts of Interest

The authors declare no conflicts of interest.

## Data Availability

The data used to support the findings of this study are included within the paper.
